# Teaching the New Ways: Improving Resident Documentation for the New 2023 Coding Requirements

**DOI:** 10.5811/westjem.21183

**Published:** 2024-09-19

**Authors:** Nathan Zapolsky, Annemarie Cardell, Riddhi Desai, Stacey Frisch, Nicholas Jobeun, Daniel Novak, Michael Silver, Arlene S. Chung

**Affiliations:** * Maimonides Medical Center, Department of Emergency Medicine, Brooklyn, New York; † Emory University Hospital, Department of Emergency Medicine, Atlanta, Georgia; ‡ New York University, Grossman School of Medicine, New York, New York; § Icahn School of Medicine at Mount Sinai, New York, New York; ∥ University of Vermont Medical Center, Burlington, Vermont

## BACKGROUND

In January 2023, significant changes to the structure of the Current Procedural Terminology CPT(R) evaluation and management (E/M) codes (here forward called the 2023 E/M changes) were implemented for emergency department (ED) encounters. These modifications aim to lessen administrative workload and accurately match coding with contemporary patient care practices.^1^ They are anticipated to impact roughly 85% of the relative value units of ED care^2^ and, thus, also have significant financial implications for EDs. Residents provide front-line care and documentation for millions of patients seen in United States EDs annually. The Model of Clinical Practice of Emergency Medicine^3^ identifies financial principles, such as billing and coding, to be required core content for board certification. Furthermore, the Accreditation Council for Graduate Medical Education (ACGME) includes quality clinical documentation to be one of the milestones that determine advancement in residency training.^4^ Interventions that alleviate documentation burden are also associated with improved physician well-being per the existing literature.^5^ However, research suggests that most emergency medicine (EM) residents do not receive formal training in billing and coding and have knowledge gaps in this area.^6^ ^–^ ^8^

Historically, documentation of encounters in the ED focused on the number of elements within history of present illness (HPI), review of systems (ROS), physical exam (PE), and medical decision-making (MDM). These new coding guidelines shift the focus almost entirely to MDM. They emphasize documentation of differential diagnoses; independent interpretation of medical testing; justification of testing not pursued; social determinants of health; chronic diseases; histories; communication with consultants, ancillary staff, and primary care; and review of external records.

## OBJECTIVES

We sought to improve resident understanding of and compliance with the 2023 E/M changes. Objectives included identification of the key elements required at each E/M level, charting and receiving feedback of sample encounters, and appreciation for the importance of accurate and high-quality documentation. We secondarily sought to investigate whether our intervention improved resident wellness specifically via benefits in confidence to perform accurate and expeditious documentation and completion of charting in a timely manner.

## CURRICULAR DESIGN

Our curriculum was developed using Kern’s six-step approach to curriculum design^9^ as a part of the educational quality improvement process at a single EM residency program based at a single, large, tertiary-care, urban hospital with an approximate annual ED patient census of 110,000 from October 1, 2022–February 28, 2023. Prior to study initiation a needs assessment was performed. Key stakeholders in departmental billing and coding were identified and interviewed, and relevant literature was reviewed.^1^ ^–^ ^10^ This included the hospital chief medical information officer, ED vice chair, and billing and coding leadership. The interviews revealed a shared opinion that often the documentation to reach the appropriate expected level of service (4 or 5) was lacking to support that level of billing and most of that documentation should be captured in the MDM portion of the note. Thus, the MDM portion of the note was targeted for the intervention.

Our educational methods primarily used in-person, flipped-classroom sessions. We decided to use a flipped-classroom approach for several reasons: 1) to allow residents to gain exposure to the new billing criteria prior to the in-person sessions; 2) as a mechanism to assess resident understanding and skills, both individually with homework responses as well as in a group setting; and 3) to use faculty’s in-person time for oversight and feedback.^11^ We also applied a spaced learning approach to maximize knowledge acquisition and retention.^12^ The sessions were held on December 14, 21, and 28, 2022.

For pre-session homework each week, all residents were provided a sample patient HPI, ROS, and PE components. All learners were provided the same case, and cases were changed each week. Cases were formulated to include elements that could be expanded upon in the MDM, Residents were also provided with the “CPT Evaluation and Management (E/M) Code and Guideline Changes” document.^10^ They were then asked to create an MDM section in accordance with the above document. Homework responses were reviewed by faculty, and feedback was given individually via email. Written feedback for residents was generated using a template based largely on the 2023 E/M guidelines changes.^10^ An ideally documented sample MDM section was also supplied for reference (Supplement 1).^13^

During each 30-minute session, residents were divided into small groups of four and provided an example patient case, which included only the HPI, ROS, and PE components. Residents then collaboratively wrote an MDM section for the case. All groups were provided the same case, and cases were changed each week to focus on different aspects of the MDM section. Each small group of residents shared their response with the larger group and were provided peer feedback under the guidance of a faculty facilitator selected for their advanced knowledge in either education or operations. Facilitators were provided in advance with an example of an ideally documented MDM section, which residents were also provided with at the conclusion of the exercise.

## IMPACT/EFFECTIVENESS

We employed a pre-post interventional study design using a convenience sample of residents, in which group assignment was based on the number of trainings each resident could attend due to scheduling factors outside the scope of this study. This study was determined to be exempt by the institutional review board of Maimonides Medical Center. Participation was voluntary and anonymous. We evaluated the impact of our brief educational intervention on subjective measures of EM resident knowledge, skills, and attitudes via survey and on objective measures of skills and behaviors by assessing aggregate chart data.

Surveys were developed through a group iterative process that included one author (ASC) with expertise in survey design methodology. RedCap,^14^ ^,^ ^15^ hosted at [Maimonides Medical Center] was used to anonymously distribute both pre- and post-intervention surveys. Both surveys consisted of six Likert-scale questions, three regarding their reported use of documentation shortcuts, and three assessing attitudes about their own understanding of and predicted skill with the new E/M coding changes. Six additional multiple-choice questions assessed knowledge about documentation rules. A final question was for feedback and requested ideas for other E/M billing and coding education. The pre-intervention survey, distributed November 30, 2022, differed from the post-intervention survey of December 28, 2022, only in asking the self-reported number of sessions attended. (Supplement 2).

Resident skills were assessed using actual clinical documentation. Resident aggregate E/M levels were assessed across three months pre-intervention (October 1–December 31, 2022) and two months post-intervention (January 1–February 28, 2023). Due to variation in resident clinical schedules, we chose the above time periods to capture the greatest proportion of the ED encounters documented by residents. We used the Kirkpatrick model to evaluate our intervention’s impact.^16^ Surveys were used to assess resident subjective reactions, and objective knowledge by identification of billable elements in a provided sample MDM. We used actual clinical documentation to assess changes in behavior. Specifically, we assessed whether trainees had a statistically significant increase (*P* < 0.05) in the proportion of E/M level 5 charts (99285) and likewise a significant decrease in level 1, 2, 3, and 4 charts (99281, 99282, 99283, 99284).

We used descriptive statistics and comparison of means with the Mann-Whitney U test stratified by number of educational sessions attended to analyze significant differences in knowledge and attitudes before and after the intervention. For knowledge, these calculations were summarized with median and interquartile range (IQR) and compared across time periods using an exact Wilcoxon signed-rank test. A Bonferroni correction for the significance of the intervention changes the alpha to 0.01667. For each chart level (99281–99285), we created logistic regression models using generalized estimating equations for individual repeated measures to account for personal variability. The number of attended flipped-classroom sessions was treated as the independent variable. Zero trainings were considered to be the pre-period for analysis. All analyses considered alpha ≤ 0.05 to be statistically significant and were conducted using SPSS v 28.0^15^ (SPSS Statistics, IBM Corp, Armonk, NY).

Forty-six of the 54 EM residents (85%) eligible for the study completed both pre- and post-intervention surveys. All 54 residents participated in at least one survey. Due to clinical schedules, some residents were not present at one or more of the three offered sessions. The first live session was attended by 33 (61%) residents, the second by 38 (70%), and the third by 40 (74%). Six (11%) residents attended one session, 15 (28%) attended two sessions, and 25 (46%) attended three sessions. Eight (15%) did not attend any sessions.

Residents demonstrated a significant improvement in knowledge regarding which elements are the key to the MDM within the 2023 E/M changes [6 (5–6.5) to 8 (7.5–8) *P* < 0.001], and by correctly identifying the number and complexity of problems, complexity of data, risk level, and the overall complexity of a sample encounter. There was no statistically significant improvement in identification of the important coding elements (4 [3–5] to 5 [3.5–5], *P* = 0.38). Residents also endorsed greater confidence in their ability to describe (2 [1–3] to 4 [3–4], *P* < 0.005), accurately document (3 [2–3] to 4 [3–4], *P* < 0.005), and bill (2 [2–3] to 3 [2–3] *P* < 0.005). There were no significant changes in their opinion of their ability to complete their charts in a timely manner (*P* < 0.19, CI 0.165–0.215) in the decision to use dictation software (*P* = 1), shortcuts (*P* = 1), or custom prepared text phrases (*P* = 1) following the intervention. Residents participating in any number of flipped-classroom sessions showed significant changes in their skills, including an increase in E/M Level 5 coded charts, and a significant decrease in Level 1, 2, and 3 coded charts (*P* < 0.005). The increase in Level 5 charts and decrease in Level 3 charts were significant after just one session (Figure). No significant change was observed in Level 4 charts.

**Figure.**
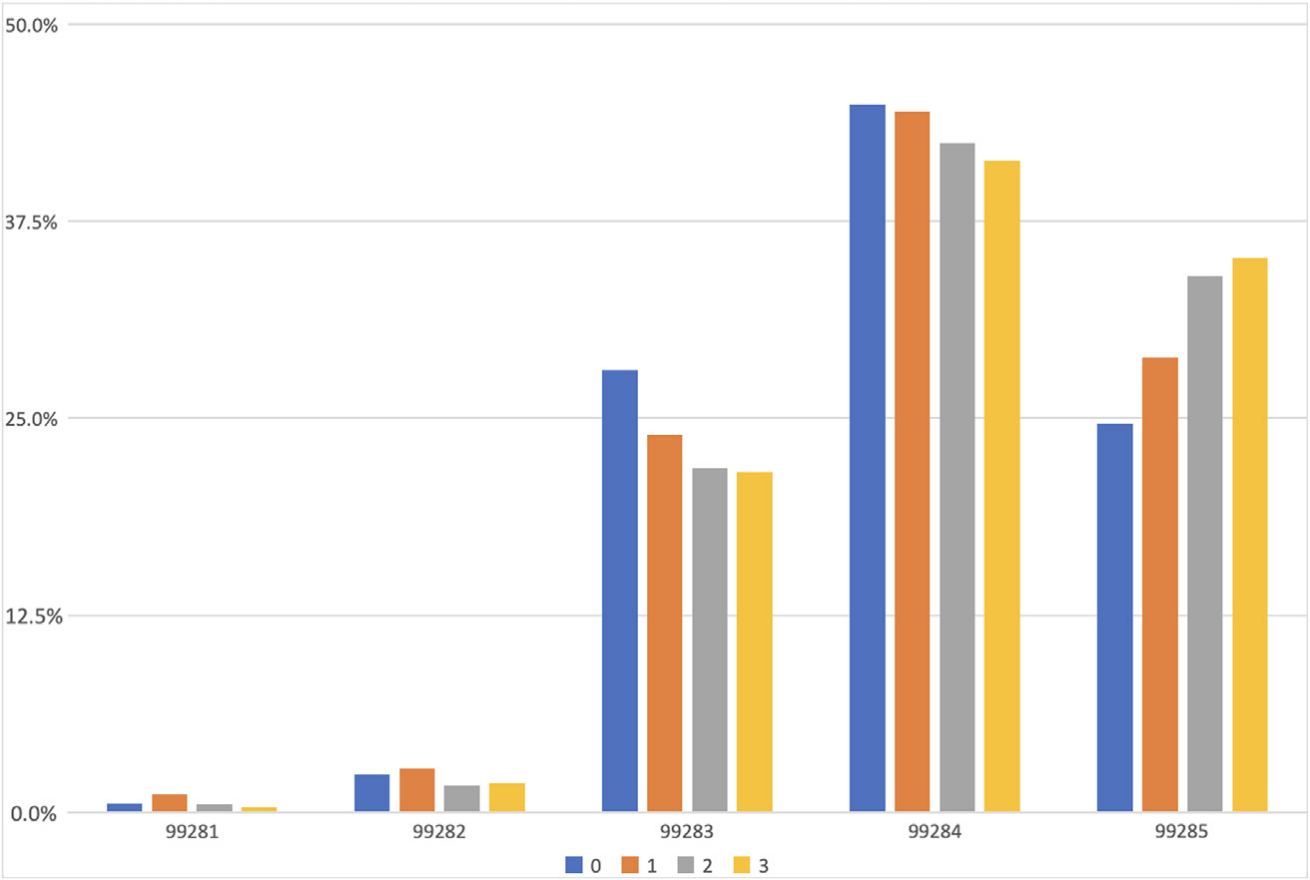
Proportion of each Current Procedural Terminology evaluation and management level by number of educational sessions attended.

To the best of our knowledge, this is the first study to date to describe the impact of an educational intervention on EM resident documentation knowledge, skills, and attitudes within the framework of the 2023 E/M changes. Naturally, our experience and results at our single EM residency program based at a large, urban, tertiary-care hospital may not be generalizable. This intervention data is single center and preliminary, and the intervention should undergo repetition and comparison before firm conclusions can be drawn.

We chose to collect data during a time range tied to the same illness season to keep the case acuity mix and attending/resident staffing comparable. We otherwise could have compared to the same months of the previous year for pre-intervention data, to best match the illness season, or alternatively, post-intervention data could have been drawn instead from the following year (2024) to help mitigate recency bias in the intervention group. That being said, the major differences in resident and attending staffing between times a year apart could also have confounded results.

We considered faculty supplemental documentation and its effect on documentation outcomes during our study design and took a pragmatic approach. For the duration of this study the attending population was stable, no significant changes to attending education were performed during this period, and attending staffing remained at baseline with no changes to ratios, shift durations, or standard distributions of encounters throughout the ED care areas. To further address this concern we attempted analysis of the attending distribution between these various groups. No attending had a greater than 1.4% change in their billing from pre- to post-intervention, and their small contributions to the overall billing for each intervention group was, therefore, unlikely to have biased the large differences seen between groups. However, the differences in distribution of attending shifts between the groups varied statistically significantly, and bias cannot be assessed without patient-level billing records. This could be considered in future studies.

Our program may have implications regarding wellness as well. Residency training must prepare emergency physicians for all aspects of their eventual professional expectations. Residents receiving education expressed greater confidence in their ability to describe, accurately document, and bill for care provided. Business literature frequently notes how a lack of clear expectations increases work stress and harms employee wellness and productivity.^18^ However, whether this association applies to emergency physicians deserves further study.

## CONCLUSION

Overall, we observed significant improvements in resident knowledge, attitudes, skills, and behaviors regarding clinical documentation. We hope to apply these successes and lessons learned to the formation of enduring education materials at our own institution for documentation improvement for both residents and attendings.

## Supplementary Information





