# Supplementary material for: Teaching the New Ways: Improving Resident Documentation for the New 2023 Coding Requirements
Source: West J Emerg Med. 2024 Sep 19;25(6):903–6. doi: 10.5811/westjem.21183 (PMC11610732; doi:10.5811/westjem.21183)
Supplement: Supplementary file 1 [file wjem-25-903-s001.docx]

**Homework Feedback Template and In-Session Practice Case**

**MDM Homework Response and Feedback**

**CC:** Chest Pain

**HPI:** 76 yo man with h/o HTN, DM, and sleep apnea who presented to the ED complaining of chest pain. He states that the pain began the day before and consisted of a sharp pain that lasted around 30 seconds, followed by a dull pain that would last around 2 minutes. The pain was located over his left chest area somewhat near his shoulder. The onset of pain came while the patient was walking in his home. He did not sit and rest during the pain, but continued to do household chores. Later on in the afternoon he went to the gym where he walked 1 mile on the treadmill, rode the bike for 5 minutes, and swam in the pool. After returning from the gym he did some work out in the yard, cutting back some vines. He did not have any recurrences of chest pain while at the gym or later in the evening. The following morning (of his presentation to the ED) he noticed the pain as he was getting out of bed. Once again it was a dull pain, preceded by a short interval of sharp pain. The patient did experience some tingling in his right arm after the pain ceased. He continued to have several episodes of the pain throughout the morning, so his daughter-in-law decided to take him to the ED around 12:30pm. The painful episodes did not increase in intensity or severity during this time. At the ED the patient was given nitroglycerin, which he claims helped alleviate the pain somewhat. -- has not experienced any shortness of breath, nausea, or diaphoresis during these episodes of pain. He has never had chest pain in the past. He has been told “years ago” that he has a right bundle branch block and premature heart beats. His daughter states her dad appeared pale last night.

**ROS:**

General: no fever, no chills, no sweats. 15 pound weight loss recently. No fatigue. Eyes/Ears/Nose/Mouth/Throat: no vertigo, no vision changes, no eye pain. No neck stiffness. Pt denies sour taste in back of throat/regurgitation. He denies reflux/heart burn.

Cardiovascular: recent chest pain-not substernal. No shortness of breath, no palpitations, no edema. No syncope.

Respiratory: occasional nonproductive cough. No hemoptysis. No wheeze.

GI: no N/V, diarrhea, blood per rectum. No abdominal pain. No change in bowel habits Genitourinary: occasionally has incomplete voiding. Some difficulty initiating urination.

MSK: rotator cuff injury to right shoulder. No pain or swelling of joints. No cramps. Neuro: no headaches. no confusion or slurred speech. No tremor. Some tingling in right arm after episode of chest pain.

Psychiatric: no depression or change in mood.

**PE:**

**Vitals:** BP 128/58 (was 147/62 at presentation to ED) HR 72; RR 12; O2 sat 97% on RA

**General:** well appearing elderly man. NAD

**HEENT:** PERRL. Clear sclera. Moist mucous membranes

Neck: supple. No masses. No thyromegaly. No bruits. No LAD

**Cardio:** RRR. S1, S2 normal without murmur/gallop/rub. No S3, S4. chest pain elicited with palpation of left chest. 2+ DP and PT pulses

**Pulmonary:** CTAB. No wheezes/rales/crackles.

**Skin:** no rash or lesions, wwp

**Psychiatry:** calm, cooperative.

**Abdomen:** soft, non-tender, non-distended. No masses. No rebound/guarding. No hepatosplenomegaly. +BS

**Extremities**: no cyanosis, clubbing, or edema.

**MSK:** decreased range of motion in shoulders. Chest pain was not elicited with movement of arms

**Neuro:** alert and oriented X3. CN II-XII grossly intact. Strength and sensation 5/5 in b/l UE and LE. Gait normal.

**Surgical Hx:**

Cervical fusion of C3-C7 with laminectomy 2000

Bilateral knee replacement 2017

**Meds:**

Hyzaar 100/25 MG QD

Furosemide 20 MG QD

Tramadol HCL 50MG QD

Ecotrin 81mg QD

**Allergies:** Penicillin: anaphylaxis/swelling of face; Scallops: anaphylaxis /swelling of face

**Family Hx:** Mom - MI at age 45, Dad - died in 70s due to heart disease

**Social Hx:** retired teacher, lives in Borough Park with wife. No smoking, or drug use. Drinks 3-4 beers/week. No recent travel.

**PMD:** Dr. Primary

**Labs**

| Na | 135 |
| --- | --- |
| K | 4.1 |
| Cl | 98 |
| CO2 | 26 |
| BUN | 21 |
| Cr | 1.2 |
| Glucose | 280 |
| CK | 143 |
| Troponin | < 0.02 |
| PT | 10.3 |
| INR | 0.9 |
| PTT | 27.5 |
| D-Dimer | 311 DDU |
| WBC | 4.6 |
| HGB | 13.4 |
| HCT | 37.8 |
| PLT | 205 |

**EKG:**


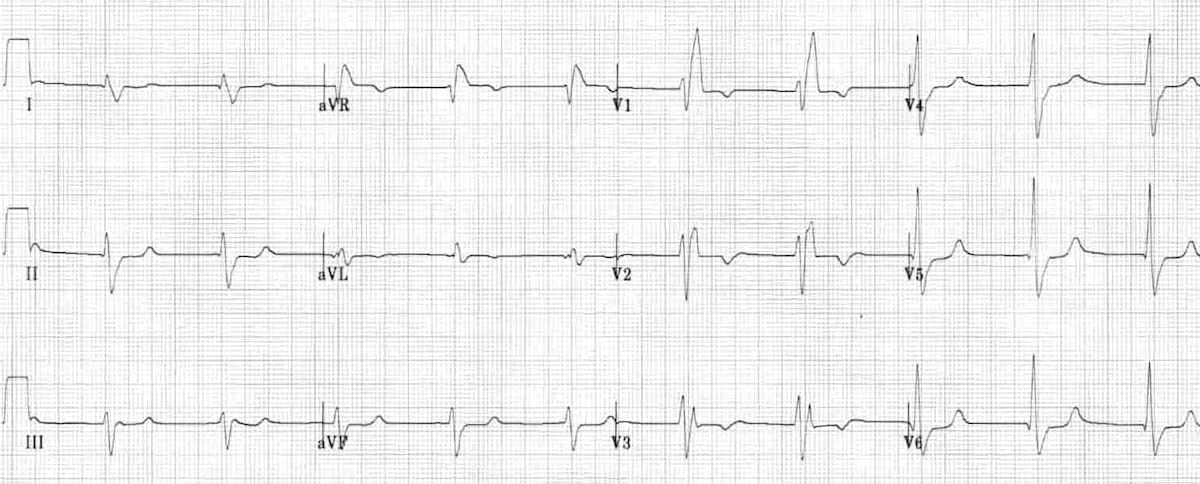


**MDM Homework Feedback**

Hey *Resident’s name*,

As you know, in an effort to be ready for the documentation changes coming January 1, we’re focusing on MDM writing this month. Overall, we were really impressed with the MDMs created for this past week’s homework, including yours. We’re giving individual feedback each week.

**The MDM you submitted was:**

*Resident’s submission here*

**Some good things you included that can help increase your RVUs/billing include:**

-Documenting comorbidities that complicate his care such as his diabetes

-Discussion of differential diagnosis

-Documenting who you obtained history from (will increase your billing if you use more than 1 independent historian such as pt’s daughter in this case)

-Discussing which tests you considered but decided not to order based on risk/benefit (for example CTA in this case)

-Providing interpretations for labs and ekgs

-Discussed patient’s care with other physicians (such as his PMD in this case)

**Some things you can include to further improve your MDM include for this example and/or IRL:**

-document DM with hyperglycemia as an additional problem

-Review of external notes if applicable

-Documenting who you obtained history from (will increase your billing if you use more than 1 independent historian such as pt’s daughter in this case). In our system this is a check box but in other EMRs it may need to be written directly in your MDM section.

As a medical note, don't forget about using theYEARS algorithm to have a higher D-dimer cutoff of 500 DDU (our hospital’s units) or 1000 FEU (other hospitals use FEU).

**Overall great work.**

*Faculty Facilitator*

PS. **Here is another sample MDM for this same case:**
76 yo man with PMH HTN, DM, and sleep apnea presents for acute chest pain radiating to the shoulder. Hx provided by pt and his daughter.

EKG RBBB (which pt states is old, though we have no priors available for comparison).

Ddx is broad, including ACS (HEART score 5, moderate risk), MSK (hx right rotator cuff injury, however pain not elicited with arm movement make this less likely), or pna/Malignancy/TB (given 15 lbs weight loss and cough).

Unlikely pneumothorax or esophageal/gi pathology given hx and PE.

Unlikely PE given pt with low risk by Wells score, no clinical e/o DVT. D-dimer is negative by YEARS algorithm so CTA is not needed for further PE ruleout.

Do not suspect dissection given HD stable, symmetric pulses. CTA not indicated at this time.

Labs with hyperglycemia

Trop negative

No leukocytosis

Hgb normal.

Plan:
-CXR

-tele monitoring

-serial ekg/trop

-Shared decision making for CDU placement given concern for possible ACS

I have discussed my findings with the pt, his family, and his PMD Dr. Primary.

**ICD Codes:**

Chest pain

DM with Hyperglycemia

Arm tingling/paresthesias
