# Supplementary material for: Teaching the New Ways: Improving Resident Documentation for the New 2023 Coding Requirements
Source: West J Emerg Med. 2024 Sep 19;25(6):903–6. doi: 10.5811/westjem.21183 (PMC11610732; doi:10.5811/westjem.21183)
Supplement: Supplementary file 2 [file wjem-25-903-s002.pdf]

Baseline Survey - Resident Documentation Skills

Thank you for taking the time to help us out!

The following survey should take no more than 10 minutes to complete. We hope to better understand the impact of a brief educational intervention on resident documentation specifically with regards to effectiveness of the medical decision making portion of the chart in light of the new CPT E/M codes effective January 1, 2023.

If you have any questions about this educational intervention or survey, please reach out to Nate Zapolsky at nzapolsky@maimonidesmed.org.

1) What is your current residency year?
- ◯ EM1
- ◯ EM2
- ◯ EM3

2) During your most recent rotation in the ED, approximately how often did you use dictation software to aid with documentation?
- ◯ Never
- ◯ Fewer than 25% of all charts
- ◯ Half of all charts
- ◯ More than 75% of all charts
- ◯ All charts

3) During your most recent rotation in the ED, approximately how often did you use tokens, dot-phrases, or other shortcuts to aid with documentation?
- ◯ Never
- ◯ Fewer than 25% of all charts
- ◯ Half of all charts
- ◯ More than 75% of all charts
- ◯ All charts

4) If you use tokens, dot-phrases, or other shortcuts, approximately what percentage were created solely by yourself (in other words, not copied from other individuals)?
- ◯ None
- ◯ Fewer than 25%
- ◯ Half
- ◯ More than 75%
- ◯ All
- ◯ I do not use any tokens, dot-phrases, or other shortcuts

5) I can describe the components of the MDM (medical decision making) section required to code for a high E/M level chart using the new coding structure beginning on January 1, 2023.
- ◯ Not at all
- ◯ A little
- ◯ Somewhat
- ◯ Well
- ◯ Perfectly

6) I will be able to effectively document patient encounters that reflect my actual clinical work using the new coding structure in the real ED setting beginning on January 1, 2023.
- ◯ Not at all
- ◯ A little
- ◯ Somewhat
- ◯ Well
- ◯ Perfectly

7) I believe my average E/M codes AFTER the new coding structure changes will be _____ than compared to my average E/M codes now, as reflected in my documentation.
- ◯ Lower
- ◯ Equal
- ◯ Higher

8) I believe my ability to complete my charts in a timely fashion AFTER the new coding structure changes will be _____ than right now.
- ◯ Slower
- ◯ Equal
- ◯ Faster

9) For the new E/M coding structure set to go live on January 1, 2023, which of the following elements of the chart have a major impact on the amount of money that can be billed for the encounter? Select all that apply.

- ☐ History
- ☐ Review of Systems
- ☐ Past Medical History
- ☐ Physical Exam
- ☐ Medical Decision Making

10) Which of the following will be key documentation elements of the chart following the new coding structure changes set to go live on January 1, 2023? Select all that apply

- ☐ Historian
- ☐ Co-morbidities
- ☐ Smoking history
- ☐ Family history
- ☐ Differential diagnosis
- ☐ Social determinants of health
- ☐ Treatments administered
- ☐ Treatments considered but not administered
- ☐ Consideration of discharge or admission

Below is an example Medical Decision Making section of a hypothetical chart. Assess the level of complexity as if you were coding for the chart yourself.

A 68 year old male with HTN, COPD and a 60 pack year smoking history presents with dyspnea, chest pain radiating to the back and left chest. History provided by EMS and patient's son. BiPAP and chained nebulizers, Mg2+, steroids, and Epi IM were used to avoid intubation. CXR interpretation on my read shows no pneumothorax. EKG shows no ST elevations or depressions and labs show borderline troponin testing and normal BNP.

11) Number and complexity of problems managed in the encounter:

- ◯ Straightforward
- ◯ Low
- ◯ Moderate
- ◯ High complexity

12) Amount or complexity of data (e.g. labs, imaging, prior records, etc.) to be reviewed and analyzed:

- ◯ Straightforward
- ◯ Low
- ◯ Moderate
- ◯ Extensive

13) Risk of morbidity from additional diagnostic testing or treatment:

- ◯ Straightforward
- ◯ Low
- ◯ Moderate
- ◯ High risk

14) Overall E/M level of MDM for this encounter:

- ◯ Straightforward
- ◯ Low
- ◯ Moderate
- ◯ High complexity

15) Just one more question (optional)! Is there anything specific on which you would like more education before these documentation changes go into effect on January 1, 2023? ____

Post Survey - Resident Documentation Skills

Thank you for taking the time to help us out!

The following survey should take no more than 10 minutes to complete. We hope to better understand the impact of a brief educational intervention on resident documentation specifically with regards to effectiveness of the medical decision making portion of the chart in light of the new CPT E/M codes effective January 1, 2023.

If you have any questions about this educational intervention or survey, please reach out to Nate Zapolsky at nzapolsky@maimonidesmed.org.

What is your current residency year?	◯ EM1<br>◯ EM2<br>◯ EM3
During your most recent rotation in the ED, approximately how often did you use dictation software to aid with documentation?	◯ Never<br>◯ Fewer than 25% of all charts<br>◯ Half of all charts<br>◯ More than 75% of all charts<br>◯ All charts
During your most recent rotation in the ED, approximately how often did you use tokens, dot-phrases, or other shortcuts to aid with documentation?	◯ Never<br>◯ Fewer than 25% of all charts<br>◯ Half of all charts<br>◯ More than 75% of all charts<br>◯ All charts
If you use tokens, dot-phrases, or other shortcuts, approximately what percentage were created solely by yourself (in other words, not copied from other individuals)?	◯ None<br>◯ Fewer than 25%<br>◯ Half<br>◯ More than 75%<br>◯ All<br>◯ I do not use any tokens, dot-phrases, or other shortcuts
I can describe the components of the MDM (medical decision making) section required to code for a high E/M level chart using the new coding structure beginning on January 1, 2023.	◯ Not at all<br>◯ A little<br>◯ Somewhat<br>◯ Well<br>◯ Perfectly
I am able to effectively document patient encounters that reflect my actual clinical work using the new coding structure in the real ED setting beginning on January 1, 2023.	◯ Not at all<br>◯ A little<br>◯ Somewhat<br>◯ Well<br>◯ Perfectly
I attended small groups during Wednesday conference on the following dates. Select all that apply.	☐ 12/14<br>☐ 12/21<br>☐ 12/28<br>☐ I did not attend small groups on any of the dates listed

Participation in small groups on 12/14, 12/21, and/or 12/28 improved my recognition of important elements to record in the medical record.	◯ Not at all<br>◯ A little<br>◯ Somewhat<br>◯ Well<br>◯ Perfectly<br>◯ I did not attend small groups on any of the dates listed
Participation in small groups on 12/14, 12/21, and/or 12/28 improved my ability to organize important elements of the clinical encounter into the medical record.	◯ Not at all<br>◯ A little<br>◯ Somewhat<br>◯ Well<br>◯ Perfectly<br>◯ I did not attend small groups on any of the dates listed
I believe my average E/M codes AFTER the new coding structure changes will be _____ than compared to my average E/M codes before the changes as reflected in my documentation.	◯ Lower<br>◯ Equal<br>◯ Higher
I believe my ability to complete my charts in a timely fashion AFTER the new coding structure changes will be _____ than before the changes.	◯ Slower<br>◯ Equal<br>◯ Faster

For the new E/M coding structure that will go live on January 1, 2023, which of the following elements of the chart have a major impact on the amount of money that can be billed for the encounter? Select all that apply.	☐ History<br>☐ Review of Systems<br>☐ Past Medical History<br>☐ Physical Exam<br>☐ Medical Decision Making
Which of the following are key documentation elements of the chart following the new coding structure changes starting on January 1, 2023? Select all that apply	☐ Historian<br>☐ Co-morbidities<br>☐ Smoking history<br>☐ Family history<br>☐ Differential diagnosis<br>☐ Social determinants of health<br>☐ Treatments administered<br>☐ Treatments considered but not administered<br>☐ Consideration of discharge or admission

Below is an example Medical Decision Making section of a hypothetical chart. Assess the level of complexity as if you were coding for the chart yourself.

A 68 year old male with HTN, COPD and a 60 pack year smoking history presents with dyspnea, chest pain radiating to the back and left chest. History provided by EMS and patient's son. BiPAP and chained nebulizers, Mg^{2+}, steroids, and Epi IM were used to avoid intubation. CXR interpretation on my read shows no pneumothorax. EKG shows no ST elevations or depressions and labs show borderline troponin testing and normal BNP.

Number and complexity of problems managed in the encounter:

◯ Straightforward
◯ Low
◯ Moderate
◯ High complexity

Amount or complexity of data (e.g. labs, imaging, prior records, etc.) to be reviewed and analyzed:

◯ Straightforward
◯ Low
◯ Moderate
◯ Extensive

Risk of morbidity from additional diagnostic testing or treatment:

◯ Straightforward
◯ Low
◯ Moderate
◯ High risk

Overall E/M level of MDM for this encounter:

◯ Straightforward
◯ Low
◯ Moderate
◯ High complexity

If you have any feedback about this educational intervention on improving documentation skills (lecture + 3 small groups), please let us know! (optional) __________________________________
